# Supplementary material for: MPCD Index for Hepatocellular Carcinoma Patients Based on Mitochondrial Function and Cell Death Patterns
Source: Int J Mol Sci. 2024 Dec 26;26(1):118. doi: 10.3390/ijms26010118 (PMC11719604; doi:10.3390/ijms26010118)
Supplement: Supplementary file 1 [file ijms-26-00118-s001.zip › Supplementary Figures.pdf]

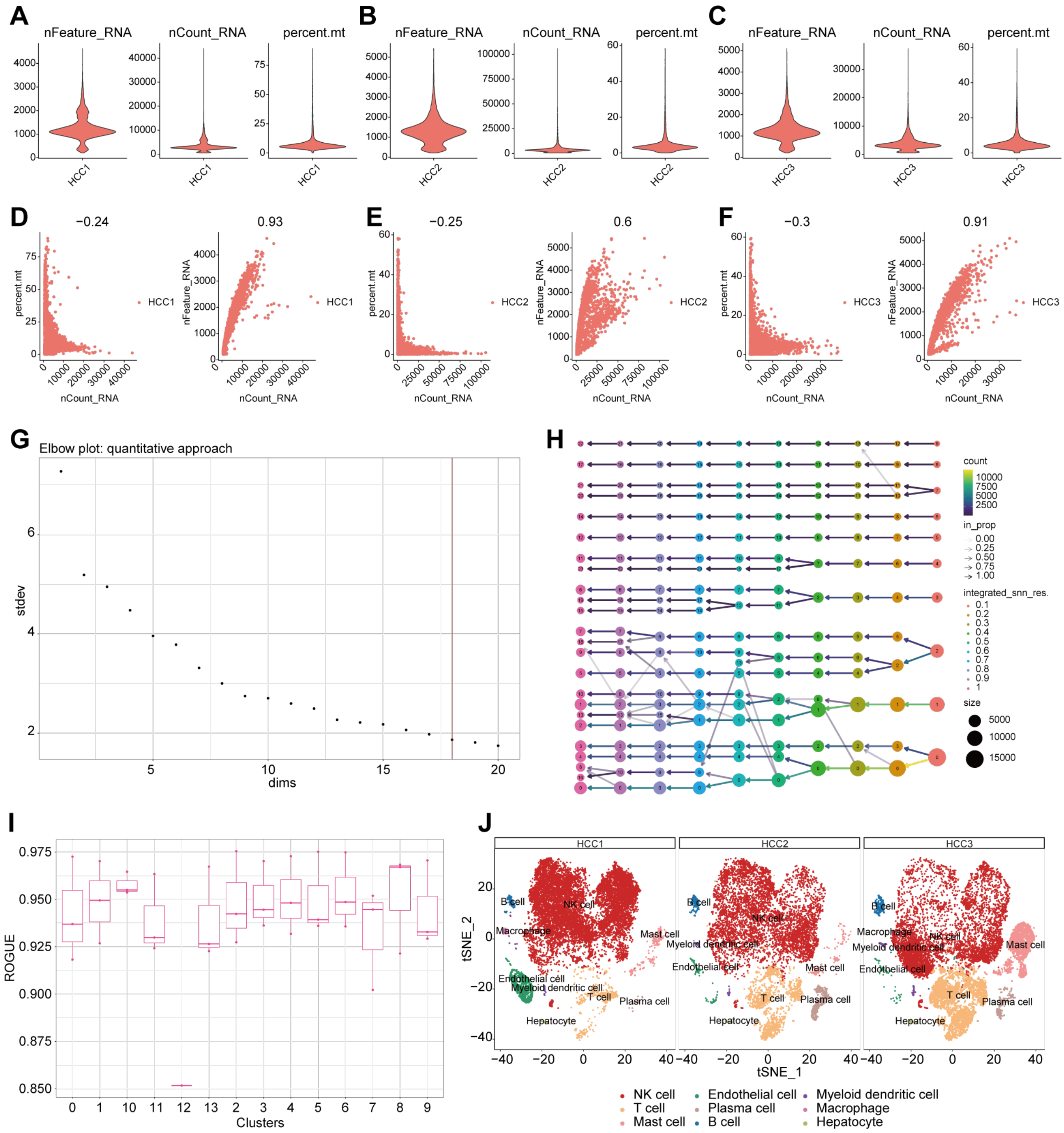

**Figure S1 Single-cell atlas of tumor tissue in HCC patients**

**A-C**. Quality control process for scRNA-Seq data from three HCC patient samples. **D-F**. Relationship between the number of genes detected, sequencing depth, and mitochondrial gene expression. **G**. Scree plot showing changes in the dimensions of dimensionality reduction. **H**. Dendrogram showing changes at different resolutions. **I**. Cluster purity calculated using the ROGUE method. **J**. t-SNE plot displaying the classification of cell subsets in 3 HCC patient samples.

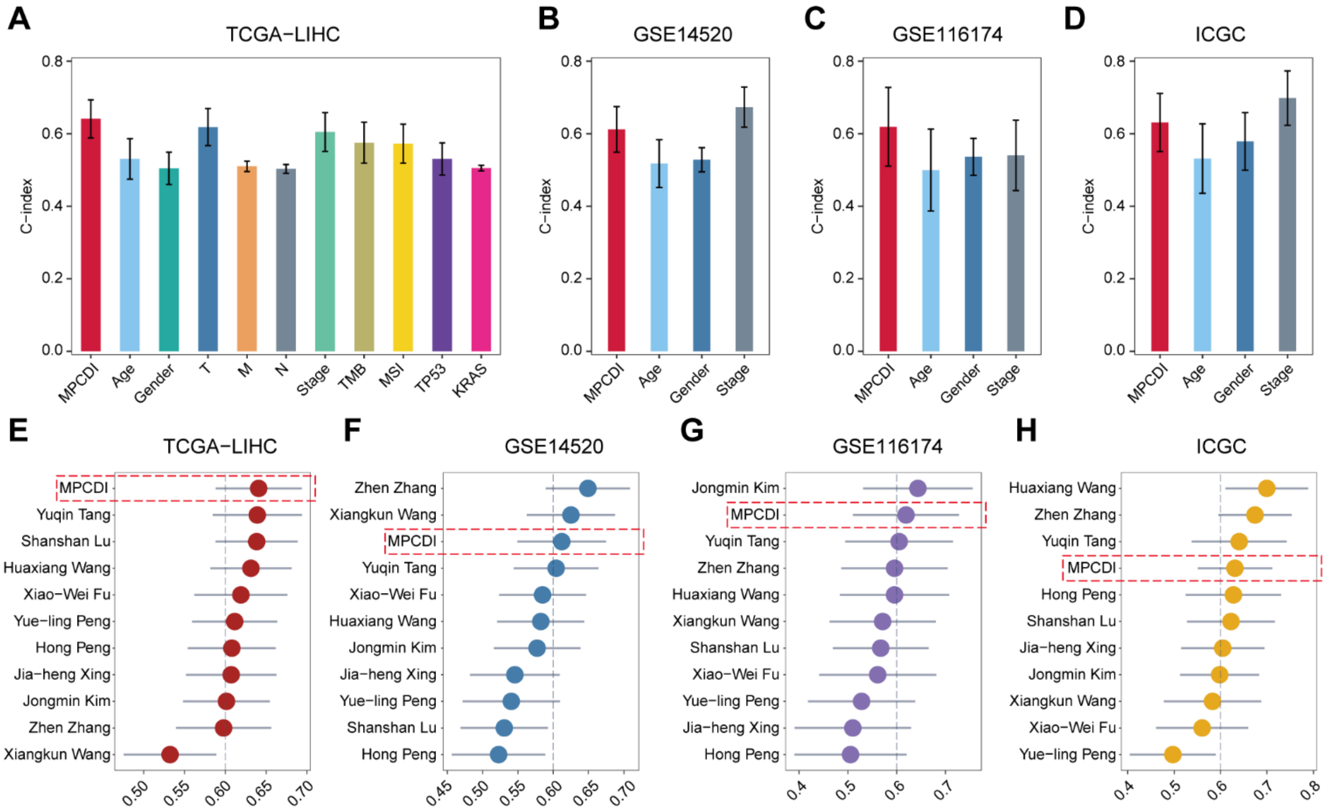


**Figure S2 Comparison of the MPCDI signature with other features or models**

**A-D**. Comparison of the C-index of the MPCDI signature with other clinical and molecular features across four datasets. **E-H**. Comparison of the MPCDI signature with ten other published model signatures across four datasets.

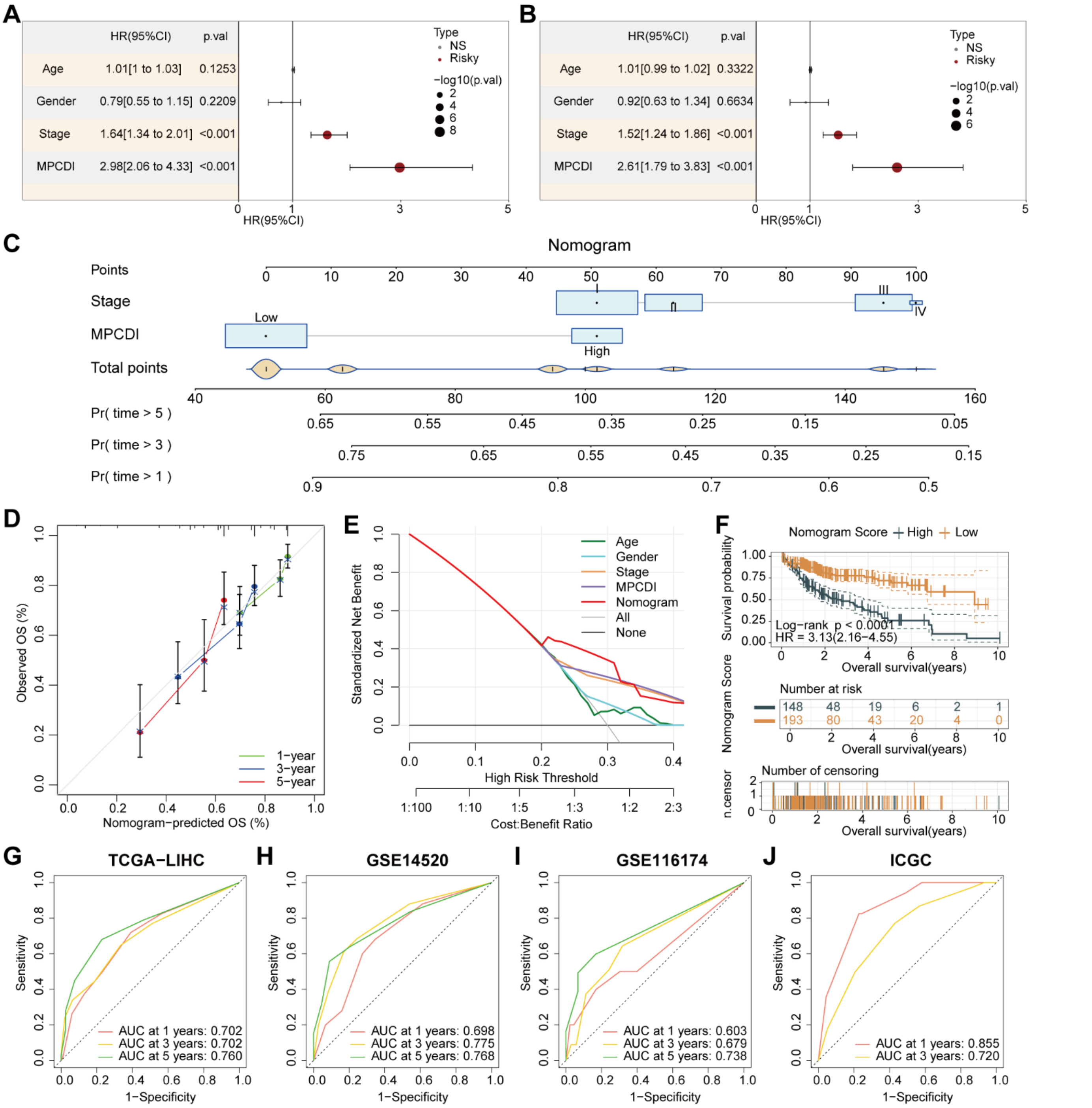

**Figure S3 Development and evaluation of the nomogram survival model**

**A**. Univariate analysis of clinical-pathological features and MPCDI subtypes in the TCGA dataset. **B**. Multivariate analysis of clinical-pathological features and MPCDI subtypes in the TCGA dataset. **C**. Construction of a nomogram to predict the prognosis of HCC patients. **D**. Calibration plots in the TCGA dataset for predicting 1, 3, and 5-year OS. **E**. DCA for predicting 1, 3, and 5-year OS. **F**. Kaplan-Meier analysis based on nomogram scores for two groups of HCC patients. **G-J**. ROC analysis for the TCGA, GSE14520, GSE116174, and ICGC datasets.

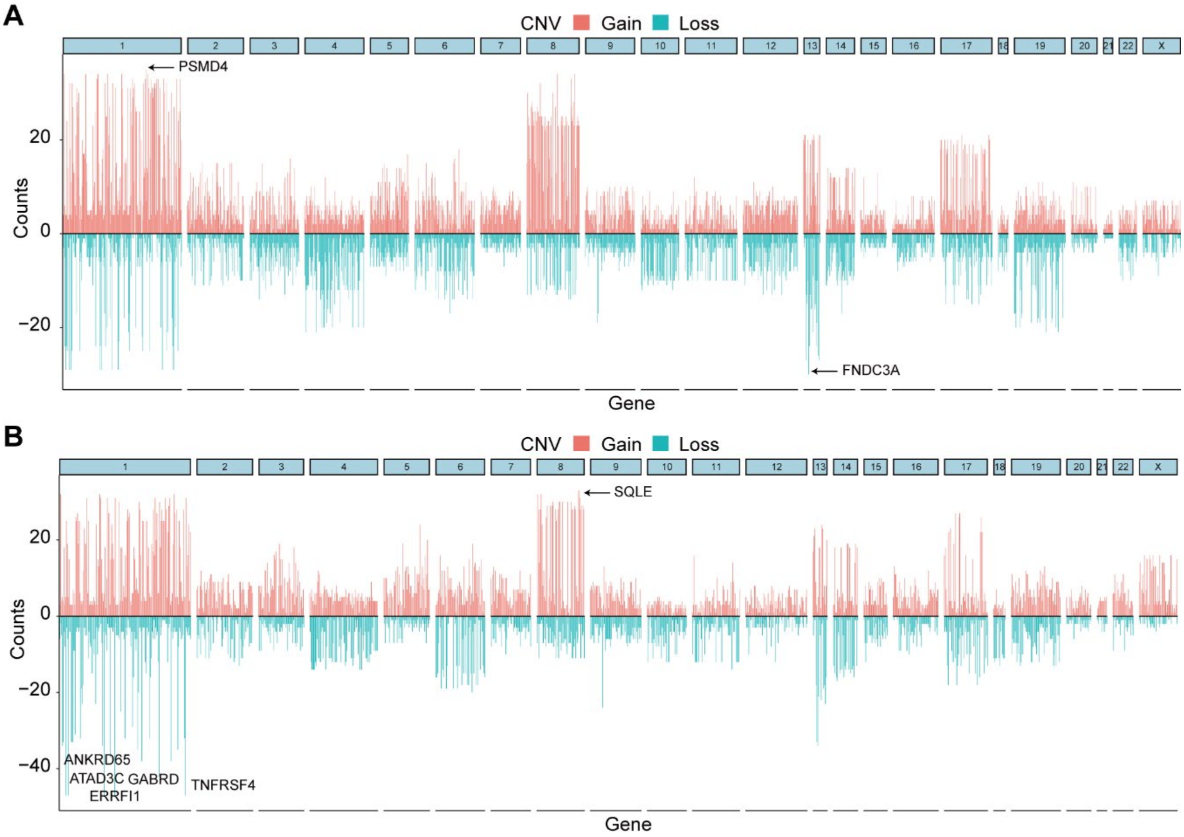


**Figure S4 CNV status analysis of MPCDI subtypes**

CNV values of differential genes in the high-MPCDI (**A**) and the low-MPCDI (**B**) subtype.

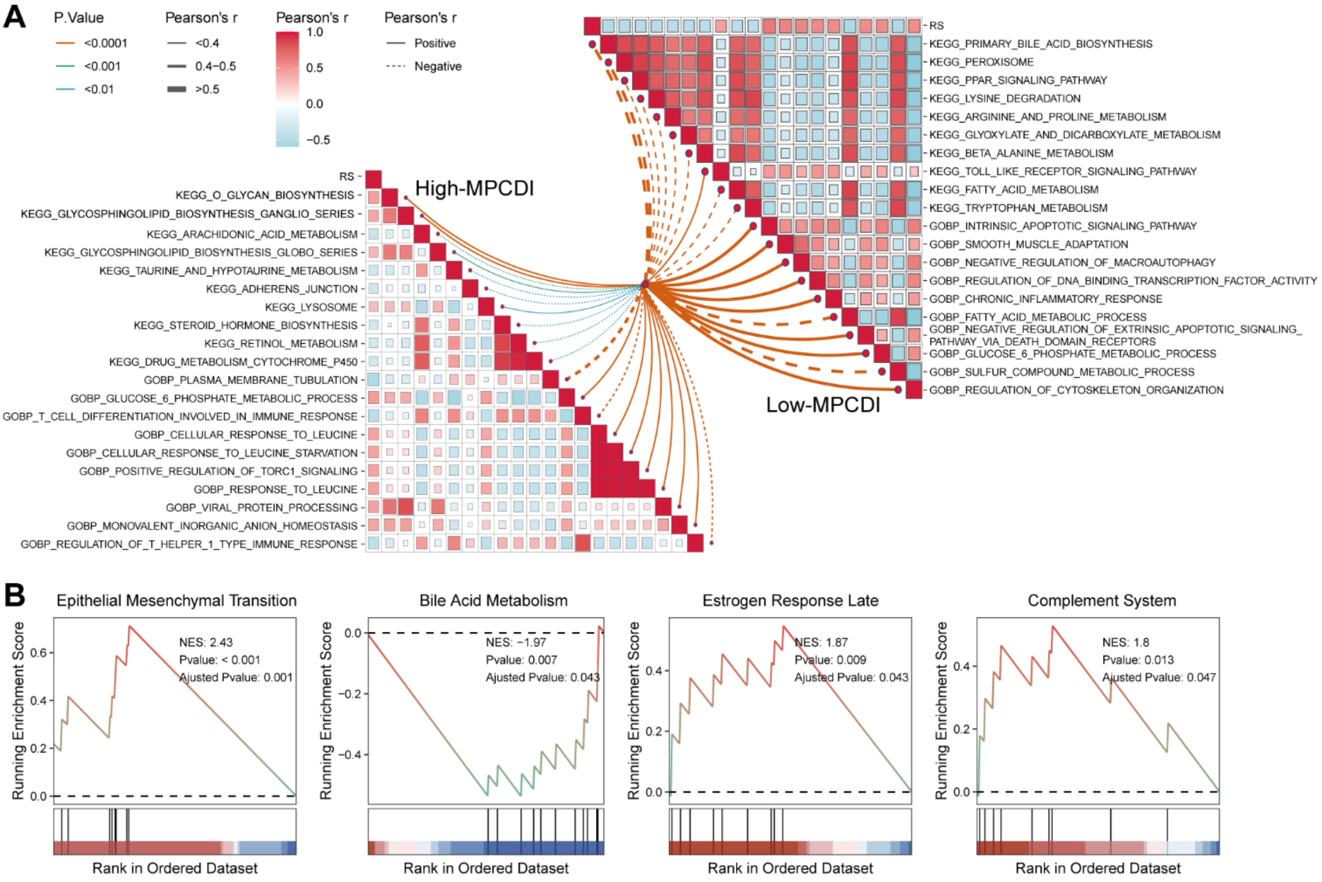


**Figure S5 Potential biological mechanisms of the MPCDI signature**

**A**. GSVA of the MPCDI signature based on GO and KEGG. **B**. GSEA of the high MPCDI subtype based on Hallmark pathways.

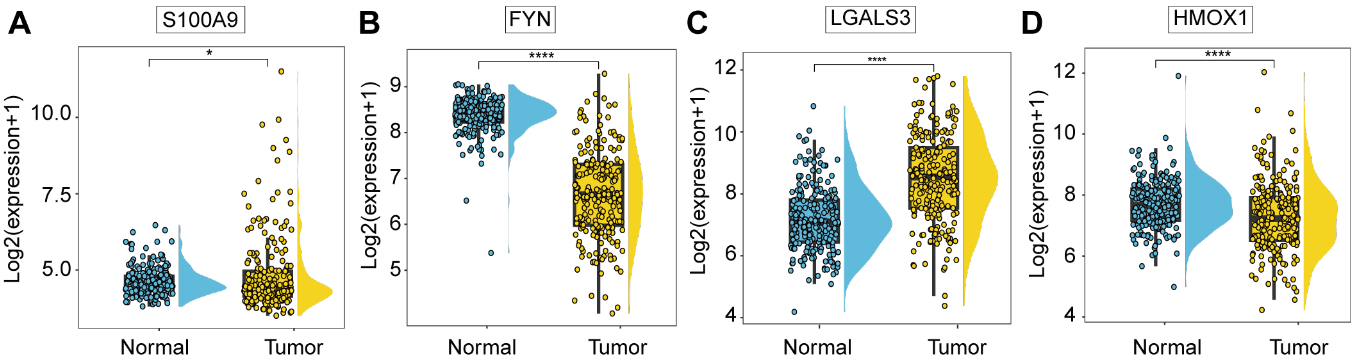


**Figure S6 RNA expression levels of four MPCDI signature genes in the GSE14520 dataset**
